# Fusion proteins with chromogenic and keratin binding modules

**DOI:** 10.1038/s41598-019-50283-0

**Published:** 2019-10-01

**Authors:** Ana Tinoco, Egipto Antunes, Madalena Martins, Filipa Gonçalves, Andreia C. Gomes, Carla Silva, Artur Cavaco-Paulo, Artur Ribeiro

**Affiliations:** 1 CEB - Centre of Biological Engineering, University of Minho, Campus de Gualtar, 4710-057 Braga, Portugal; 2 Centre of Molecular and Environmental Biology (CBMA), Department of Biology, University of Minho, Campus of Gualtar, 4710-057 Braga, Portugal

**Keywords:** Protein design, Bioinspired materials

## Abstract

The present research relates to a fusion protein comprising a chromogenic blue ultramarine protein (UM) bound to a keratin-based peptide (KP). The KP-UM fusion protein explores UM chromogenic nature together with KP affinity towards hair. For the first time a fusion protein with a chromogenic nature is explored as a hair coloring agent. The KP-UM protein colored overbleached hair, being the color dependent on the formulation polarity. The protein was able to bind to the hair cuticle and even to penetrate throughout the hair fibre. Molecular dynamics studies demonstrated that the interaction between the KP-UM protein and the hair was mediated by the KP sequence. All the formulations recovered the mechanical properties of overbleached hair and KP-UM proved to be safe when tested in human keratinocytes. Although based on a chromogenic non-fluorescent protein, the KP-UM protein presented a photoswitch phenomenon, changing from chromogenic to fluorescent depending on the wavelength selected for excitation. KP-UM protein shows the potential to be incorporated in new eco-friendly cosmetic formulations for hair coloration, decreasing the use of traditional dyes and reducing its environmental impact.

## Introduction

Peptides and proteins have attracted great attention in cosmetic industry for the development of new cosmetic formulations. Particularly, fusion proteins can be used on the development of advanced cosmetic products. Combining more than one function in the same protein sequence, we can explore these proteins for several applications, which represents a huge advantage comparing to the current cosmetic products^1^. Fusion proteins are already used in the cosmetic industry, mainly for skincare. A formulation composed by human serum albumin fused with cytokine peptides was developed for improving skin appearance, smoothing wrinkles and whitening skin^2^. Also, a skin-penetrating peptide was fused with a physiologically active protein to promote collagen synthesis and improve skin conditions^3^.

Chromogenic proteins are colored proteins that could be obtained from reef building corals of the class Anthozoa or the sea anemone, *Actinia equine*, or simply synthetically produced from green fluorescent protein (GFP)^4^. The basic structure of the chromogenic proteins is identical to the one of GFP, and is composed by eleven antiparallel β-strands forming a β-barrel that surrounds and protects the α-helix structure of the chromophore. The environment of the chromophore is of high importance, since it can affect the protein spectral properties by influencing chromophore position, protonation state, and electron delocalization^5,6^.

One example of chromogenic protein is the monomeric and non-fluorescent Ultramarine (UM) protein, derived from Rtms5 which presents an intense dark blue color. Rtms5 protein was isolated from the reef building coral *Montipora efflorescens* and 5 different genetic mutations were introduced to obtain the monomeric UM protein: V44A, S125R, F162R, L127T and H146N^7^. In this work, we explored UM chromogenic properties to color and protect overbleached hair.

Human hair is a complex fiber divided into cuticle, cortex, and medulla; and its composed of dead cells mainly filled with keratin and other constituents like water, lipids, pigments and trace elements^8^.

Hair, besides conferring protection, is also a symbol of beauty and wealth with significant socio-economic impact^9^. Cosmetic industry has developed several processes and products to improve hair’s health, style, feel, shine and color. Although a desired look can be obtained, some of these products and processes can be aggressive to the hair leading to the degradation of hair’s structure and decrease of hair’s mechanical properties^8^. The most popular hair cosmetic treatments used are bleaching and permanent waving, where hair color and shape can be respectively modified. Hair coloring is an extremely successful hair care treatment which can be classified as permanent, semi-permanent or temporary coloring depending on the type of dye and methodology used^8,9^.

Different disadvantages are associated with the use of dyes in hair coloration. It can damage the hair cuticle, the cortex or even the medulla, affecting the mechanical properties of hair; and can cause allergic reactions and even skin irritation. Also, the introduction of dye-containing effluents into the wastewater treatment systems are considered a factor of environmental pollution, not only because of their color, but also because many of the dyes and related compounds (i.e. dyes breakdown products, pigments, dye intermediates, auxiliary chemicals and heavy metals) are toxic, carcinogenic or mutagenic to life forms. Adequate treatments are necessary to these effluents in order to decrease the lifetime of the dyes in the environment and minimize the negative side-effects^10^.

Some solutions were already described to avoid hair fiber degradation, color fade overtime and to increase dye affinity towards hair. For example, carbon nanotubes can be chemically or physically modified by some agents like polymers and hair dyes to increase the affinity of these to the hair fiber^8,11,12^. Peptides with high affinity to hair and resistant to shampoo washing were already described. These peptides could be used as linkers between the hair and the color agent, increasing the binding efficiency of the dye to the hair fibers and maintaining the color for longer time^13^.

Concerns about possible health risks and detrimental environment effects are a major driving force for the substitution of synthetic organic compounds for hair dying by natural colorants. Also, the increasing popularity of a more natural lifestyle also stimulate the development and research on natural compounds for dying and other possible applications^14^.

In this work, UM chromogenic protein was conjugated with a keratin-based peptide (KP) to improve its affinity towards human hair^15^. The ability of the KP-UM construct to color overbleached Asian hair and recover hair’s mechanical properties was evaluated. The effect of different cosmetic formulations on hair coloration with the KP-UM protein, was also assessed in terms of color intensity, penetration degree and leaching of protein from the hair fiber.

## Results

### Purity and size of KP-UM protein

**Figure 1:**
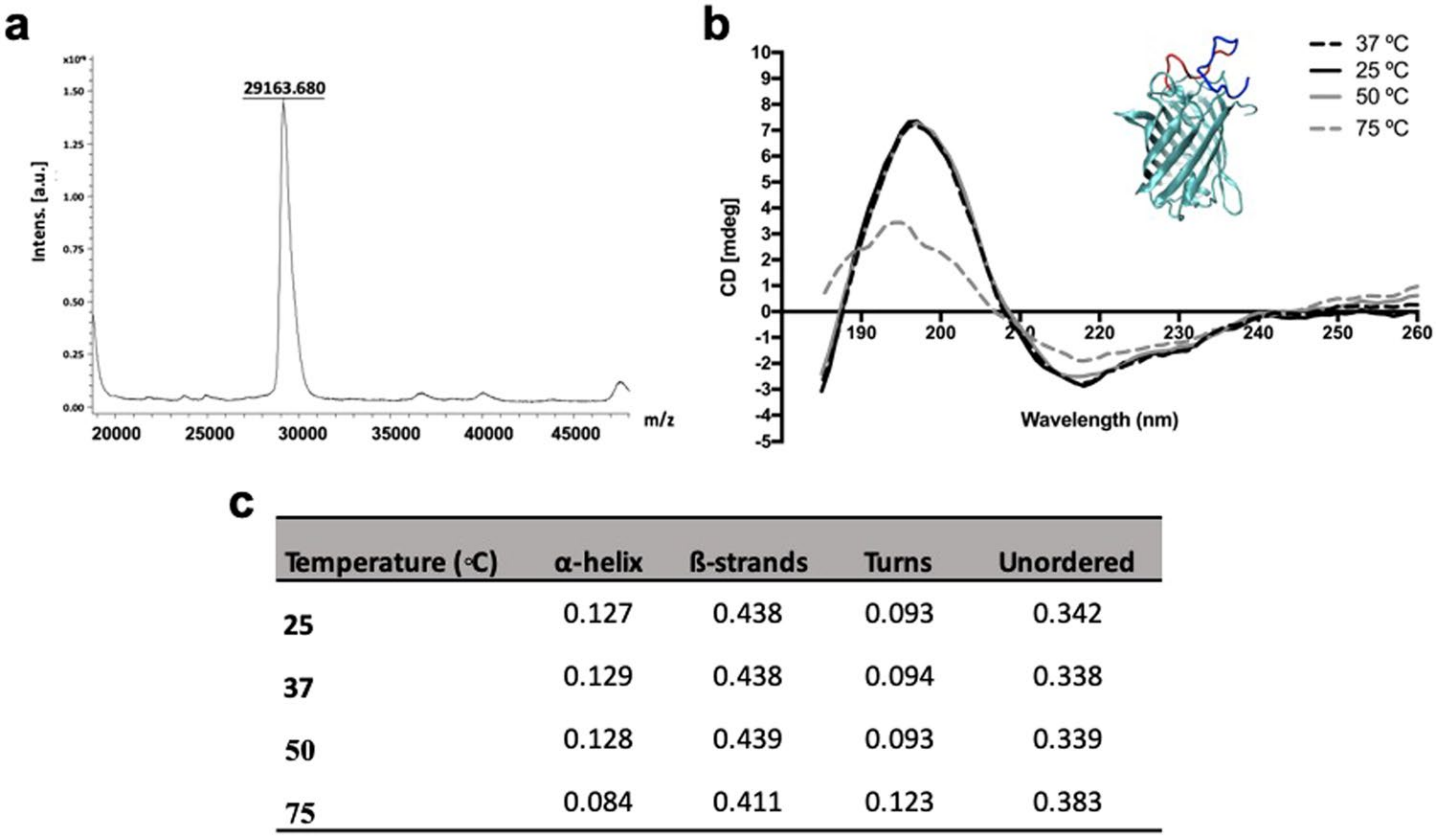
(**a**) MALDI-TOF mass spectra of KP-UM protein obtained in reflective positive mode using sinapic acid as matrix; (**b**) Circular dichroism spectra of KP-UM at different temperatures (37, 25, 50 and 75 °C): a close-up of KP-UM secondary structure is highlighted where cyan represents the UM protein, dark blue the KP sequence and red the (GA)_5_ linker; (**c**) Effect of temperature on KP-UM secondary structure determined by CD.

The amino acidic sequence of UM protein was conjugated with a keratin-based peptide (KP) using a linker composed by 5 repetitions of glycine and alanine (GA)_5_. KP-UM protein was characterized regarding purity and molecular weight after expression and purification. SDS-PAGE confirmed the high purity of KP-UM protein, presenting a single band with a molecular weight close to the theoretical (KP-UM plus the extra amino acidic sequence (His-tag) from the pet28a( + ) vector) – 29.049 kDa (data not shown). MALDI-TOF mass spectrometry (Fig. 1a) also confirmed the purity, the monodisperse character and the size of the KP-UM protein.

### Structural analysis by CD spectroscopy

To further study the effect of KP peptide and (GA)_5_ linker on Ultramarine (UM) structure^7^ and understand the role of temperature on the secondary structure of KP-UM protein, the far-UV CD spectra of this protein under different temperatures was monitored. KP-UM secondary structure was studied between 185 and 260 nm, since the π* and π to π* transitions of α-helices and β-sheets normally occur in the UV region^16^.

For this study, different temperatures were studied (Fig. 1b,c): 25 °C correspond to the average air temperature in hairdressers salons, 37 °C correspond to the body temperature and 50 and 75 °C were selected taking in consideration the procedures performed on hair styling where higher temperatures are applied.

KP-UM protein was constructed considering a GFP-like chromogenic blue protein termed Ultramarine (UM), which is derived from the naturally occurring fluorescent protein Rtms5. GFP-like proteins belong to the proteins with β-barrel topology, where the proteins resemble in an 11 stranded β-sheets wrapped around a single central helix, in the middle of which is the chromophore^17^. Analyzing the shape of KP-UM spectra in Fig. 1b, it was observed a maximum and minimum peak at 197 and 220 nm and a similarity between the spectra at 25, 37 and 50 °C. When the temperature increased to 75 °C, a decrease in the spectra intensity and a 2 nm shift of the 197 nm peak to 195 nm, was observed. To better quantify the structural transition at different temperatures, the far-UV CD spectra of KP-UM protein were further de-convoluted using the software available from the DICROWEB website. The results of secondary structure content of KP-UM protein incubated at different temperatures are listed in Fig. 1c. The values for helix 1 and helix 2 as well as strand 1 and strand 2 were added to obtain total α-helix and total β-strands content. Almost no differences were verified on the proportions of KP-UM secondary structures when the protein was incubated at 25, 37 and 50 °C. However, when the temperature was increased to 75 °C, a decrease in the α-helix and β-strands content was observed, with a concomitant increase in the content of turns and unordered structures.

### KP-UM binding to overbleached Asian hair

To evaluate the KP-UM protein capacity to bind and to color OB Asian hair, different KP-UM solutions were prepared and the binding efficiency and the color coordinates of colored hair were determined. KP-UM was dissolved in 0.1 M phosphate buffer, pH 7, or in 0.1 M phosphate buffer, pH 7, with 1.5% (v/v) propylene glycol, 0.5% (v/v) benzyl alcohol and 1 or 10% (v/v) of ethanol. The protein solutions were labelled as 0, 1 and 10% EtOH. These formulations were selected to stimulate protein interaction with the hair fiber by promoting hair swelling and exposing the hair shaft to the protein solution^18^.

**Figure 2:**
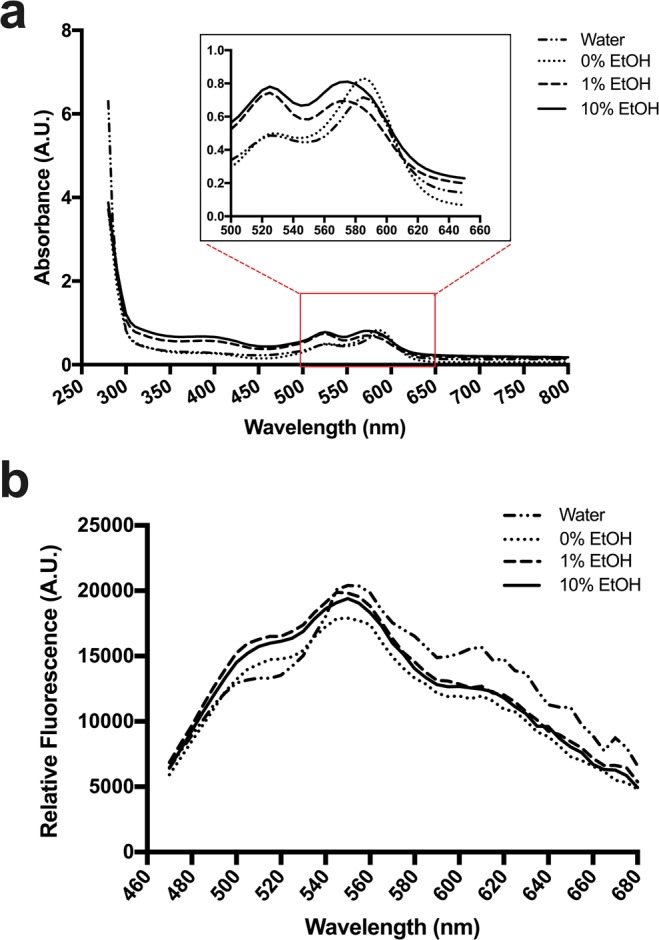
(**a**) KP-UM absorbance spectra with a close up of the absorbance peaks in the region between 500 nm and 650 nm and (**b**) KP-UM emission spectra when excited at 440 nm. KP-UM protein was prepared in different solutions: formulation with 10% ethanol (10% EtOH), formulation with 1% ethanol (1% EtOH), phosphate buffer pH 7 (0% EtOH) and water.

When preparing the KP-UM solutions, differences were observed in the color depending on the formulation where KP-UM was dissolved. To study the effect of formulations on color, the absorbance spectrum of KP-UM in each formulation was plotted and analyzed (Fig. 2a). A control of KP-UM dissolved in water was also performed. For all the formulations, two absorption peaks around 525 and 585 nm were verified, corresponding to the *trans* and *cis* conformation, respectively.

Comparing the spectra of KP-UM protein in the different formulations, it was observed a shift of the absorption peaks for lower wavelengths as the percentage of ethanol increased. For the KP-UM dissolved in water or phosphate buffer, the maximum absorption peaks were at 525 and 585 nm. When the protein was dissolved in the formulation with 1% ethanol, there was a shift of the less energetic peak from 585 to 575 nm; while there was a shift of both peaks to lower wavelengths (520 and 575 nm) when KP-UM was dissolved in the formulation with 10% ethanol. Also, the ratio between the two absorption peaks showed to be sensitive to different factors, like the solvent polarity. The differences in the ratio were verified on the obtained data, where a more intense peak at 585 nm was observed when the protein was dissolved on water or in the formulation with 0% ethanol. When ethanol was added, it was verified an alteration in the ratio of these two absorption peaks, with an increase in lower wavelength peak intensity.

Although the UM protein is classified as a chromogenic and non-fluorescence protein, the KP-UM fluorescence spectrum was determined (Fig. 2b). Initially, the KP-UM protein was excited at ${{\rm{\lambda }}}_{Abs}^{max}$ observed in the absorbance spectrum (Fig. 2a): 525 and 585 nm for KP-UM aqueous solution. However, when KP-UM was excited at these two wavelengths, no bands were observed on the emission spectrum (see Supplementary Fig. S1). Taking in consideration works performed with other GFP-like proteins and with Rtms5 mutants^19^, we tested different excitation wavelengths. When the protein was excited at 440 nm a broad emission spectrum was obtained and, although without any defined peak, three projections ${{\rm{\lambda }}}_{Em}^{max}$ around 510, 550 and 610 nm were observed.

**Figure 3:**
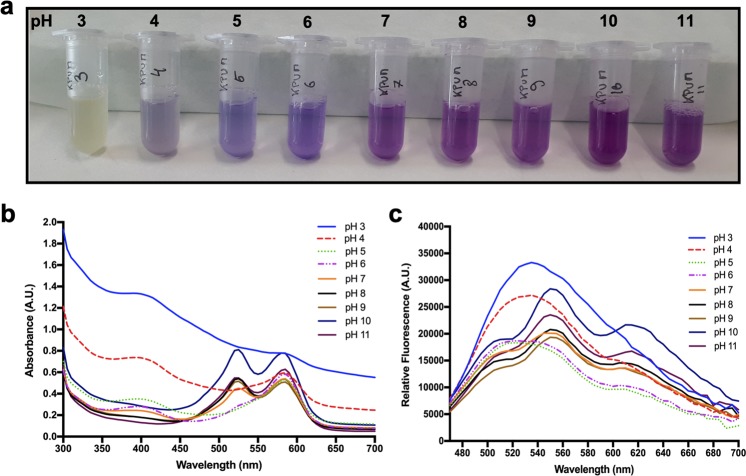
Effect of pH on (**a**) the visible color of KP-UM protein on 0.5 M phosphate solution; (**b**) absorption spectra of KP-UM protein; (**c**) fluorescence spectra of KP-UM protein.

To better characterize the KP-UM protein, the effect of the pH on the absorbance spectrum was studied between pH 3 and 11. We found that the color of KP-UM protein was pH-dependent being this color change reversible (Fig. 3a). KP-UM color can change from yellow to intense purple by increasing the pH from 3 to 11, returning to yellow by a simply decrease of pH until 3. To understand these alterations in color, each solution was studied relatively to its absorbance spectrum (Fig. 3b). The spectra for the solutions between the pH 3–5 presents only one peak at 585 nm, indicating that the *cis* conformation is favored for pH between 3 and 5. The *trans* conformation start to appear for pH 6, becoming more pronounced increasing the pH of solutions. The effect of pH on KP-UM fluorescent spectra was also studied (Fig. 3c) and, generally, two different profiles were obtained. A broad spectrum with a maximum around 530 nm was observed for the solutions with a pH between 3–6. For the other pH values, the spectra presented three peaks around 510, 550 and 610 nm, which became more pronounced for the highest pH values (10 and 11).

**Figure 4:**
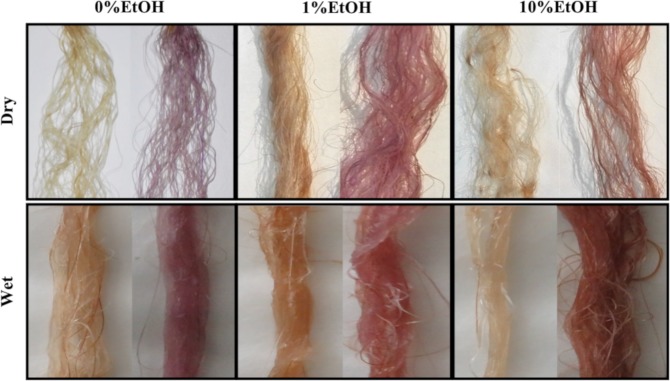
Overbleached Asian hair colored with KP-UM protein (right) and the respective controls (left). The coloration process consisted in: 0.2 g of OB Asian hair incubated with 10 mL of KP-UM formulations, for 30 min, at room temperature, and then dried for 20 min at 60 °C; this process was repeated three times.

Overbleached Asian hair was colored with the KP-UM protein dissolved in the three formulations. The hair strands were washed with commercial shampoo, 24 h after treatments, to remove roughly bound proteins (Fig. 4). All hair strands treated with the KP-UM protein acquired a purple/pink coloration supporting the ability of KP-UM to bind and colorize the hair. The hair color obtained after KP-UM treatment was dependent on the formulation where the protein was dissolved.

The binding efficiency of KP-UM was determined by quantifying the amount of protein on solution after hair incubation. A binding efficiency of 43.11 ± 0.58, 59.50 ± 0.30 and 52.79 ± 1.44% for the 0,1 and 10% EtOH formulations, was verified.

### Hair coloration degree

To evaluate the effect of formulations on hair color, CIELAB color measurements were performed to determine color coordinates, brightness, color differences and color strength by means of K/S determination.

**Figure 5:**
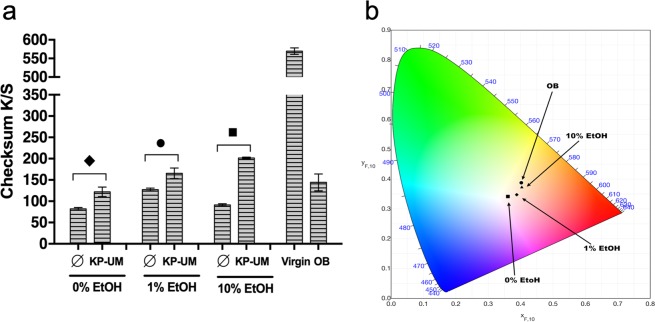
(**a**) K/S values (checksum: 360–700 nm) of hair samples obtained after incubation with KP-UM: 0% EtOH - overbleached hair incubated with 2 mg/mL KP-UM in 0.1 M phosphate buffer, pH 7; 1% EtOH - overbleached hair incubated with 2 mg/mL KP-UM in a 1% ethanol formulation; 10% EtOH - overbleached hair treated with 2 mg/mL KP-UM in a 10% ethanol formulation. Values are the mean of SD of three independent measurements. Statistical significant differences from the respective control are indicated as: *p-value ≤ 0.05, ●p-value ≤ 0.01; ♦p-value ≤ 0.001; ■p-value ≤ 0.0001; (**b**) CIE chromaticity diagram and chromaticity coordinates D_65/10_ (X_F,10_, Y_F,10_) of: ● - overbleached Asian hair; ■ - overbleached hair colored with KP-UM in 0.1 M phosphate buffer, pH 7 (0% EtOH); ◆ - overbleached hair colored with KP-UM in formulation with 1% EtOH; ▲ - overbleached hair colored with KP-UM in formulation with 10% EtOH.

Color strength of hair strands treated with KP-UM was evaluated taking into consideration the checksum of K/S values between 360 and 700 nm (Fig. 5a). The checksum of K/S values of overbleached Asian hair treated with each formulation, untreated virgin and overbleached Asian hair were also considered.

The incubation of overbleached Asian hair with the three formulations, without the KP-UM protein, resulted in a decrease in the checksum of K/S values, when compared with the untreated overbleached hair.

Significant differences in the K/S checksum values were observed when the hair was incubated with the KP-UM protein. The increase in the K/S checksum values were dependent on the amount of ethanol present on the formulation, with the highest results being observed for the hair colored with KP-UM in the formulation with 10% ethanol.

Figure 5b shows the corresponding chromaticity points D_65/10_ (X_F,10_, Y_F,10_) of the overbleached Asian hair with and without the KP-UM protein in the Commission International de l’Eclairage (CIE) diagram. This diagram maps color in terms of hue and saturation and the boundary of the CIE diagram is defined by the saturated hues that the human eye can detect (from 380 to 780 nm)^20^.

The addition of ethanol into the KP-UM formulation lead to a color change from the purple zone of the diagram (0% EtOH) to pink (1% ethanol) and even red for the highest ethanol percentage (10% EtOH). As expected, the overbleached hair is located closer to the area corresponding to the yellow color.

**Figure 6:**
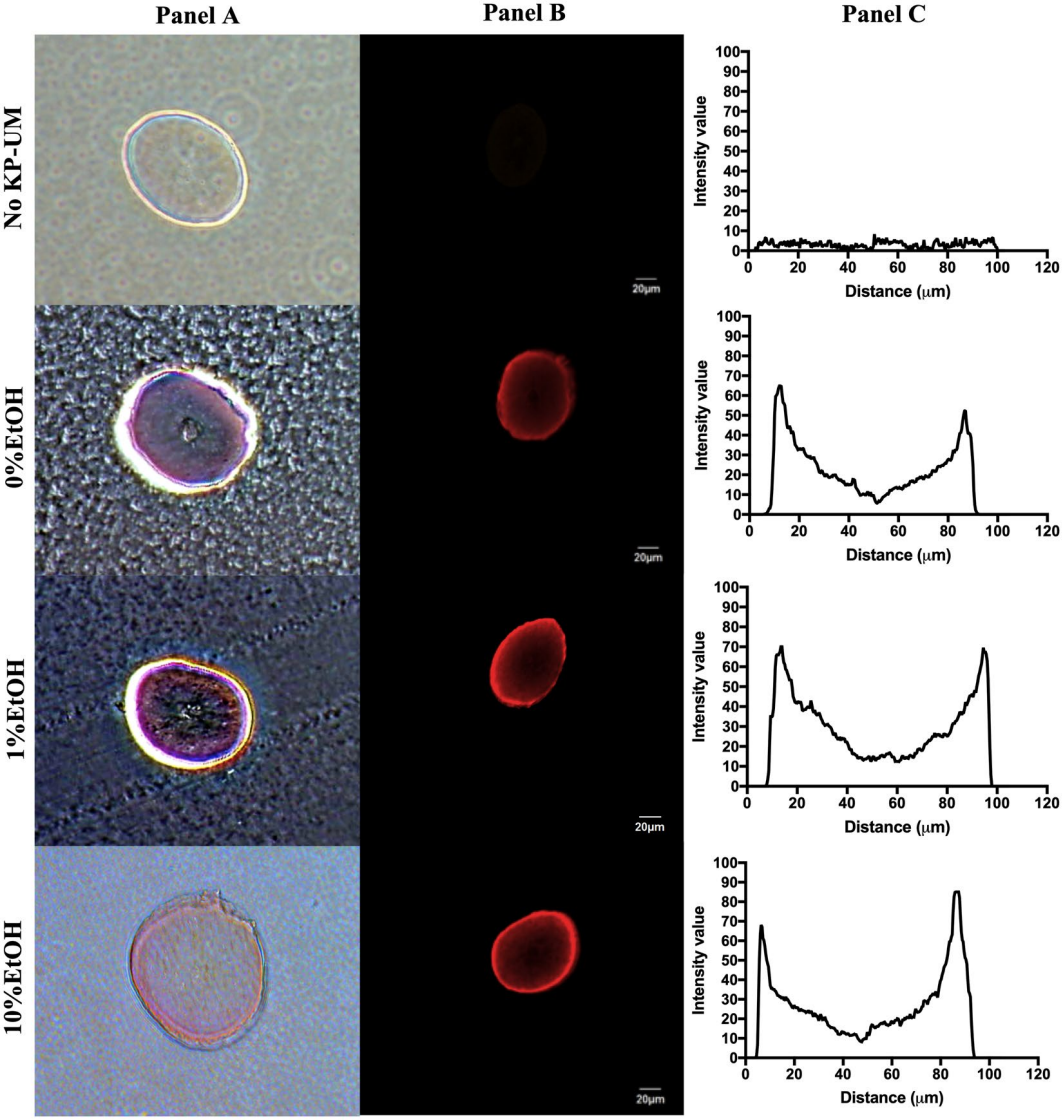
Optical microscopy (Panel A) and confocal microscopy (Panel B) of transversal cuts of overbleached Asian hair without treatment (No KP-UM) or treated with 2 mg/mL KP-UM dissolved in: 0.1 M phosphate buffer (0% EtOH), 1% ethanol formulation (1% EtOH), and 10% ethanol formulation (10% EtOH). On Panel C are represented the KP-UM penetration profiles on Asian hair’s cross sections.

To better understand the differences observed in the color of hair, and if these could be also related with the localization and penetration degree of the KP-UM protein into the hair fiber, transversal cuts of colored hair were analyzed by optical microscopy (Fig. 6 – Panel A) and by confocal microscopy (Fig. 6 – Panel B). Independently of the formulation, the KP-UM protein was able to bind to the hair and penetrate into the hair cortex. Despite that in the optical microscopy images (Fig. 6 - Panel A) the KP-UM seems to be more concentrated near the cuticle, the higher sensitivity of fluorescence microscopy show that the protein was able to bind and penetrate into all the areas of the hair cortex (Fig. 6 - Panel B).

KP-UM penetration profiles were determined taking in consideration the KP-UM relative intensity through a fiber’s cross section. Penetration profiles represented on Fig. 5 – Panel C, give an indication how the KP-UM protein distributes along the hair and if there is some tendency to accumulate in a specific area of the hair fiber. It was observed for all the samples, an intensity higher than the overbleached hair (No KP-UM) throughout the fiber cross section, mainly in the first 10–15 µm of the hair fiber.

### Recovery of the mechanical properties of overbleached hair colored with KP-UM protein

Systematic exposition of hair to chemical treatments affects irreversibly hair’s characteristics like smoothness, shining and even its mechanical properties. The mechanical resistance of overbleached Asian hair before and after treatment with the KP-UM was studied to determine the effect of the protein in hairs’ mechanical properties in terms of Young’s modulus and tensile strength (see Supplementary Fig. S2). Considering the 8 overbleaching cycles applied to the Asian hair, a loss of stiffness of about 21.5% and a loss of tensile strength of about 23.1% was verified, comparatively to the virgin Asian hair. These results agreed with the data previously reported by Tinoco *et al.*, 2018^21^.

The percentages of recovery of the hair samples colored with the KP-UM protein were determined comparatively to the respective controls and are shown on Table 1. These percentages were calculated relatively to the hair samples incubated with the respective formulations without the KP-UM protein. Analyzing Supplementary Fig. S2 and the data from Table 1, it is possible to observe that KP-UM protein in the three formulations has the capacity to recover the hair’s mechanical properties. The condition that most increased the hair’s Young's modulus was the KP-UM protein in the formulation with 0% EtOH (24.17%), while the condition which most contributed to an increase of the tensile strength was the KP-UM in the formulation with 10% EtOH (19.85%). Generally, considering the tensile strength and the Young's modulus, the best condition was the KP-UM in the formulation with 10% EtOH.

**Table 1:** Recovery (R %) of overbleached Asian hair mechanical properties measured in terms of Young’s modulus and Tensile Strength (%) after treatment with KP-UM protein.

	% Recovery	
Ethanol (%)	Young’s modulus (%R)	Tensile Strength (%R)
0	24.17	1.43
1	11.35	2.78
10	17.02	19.85

The hair samples were compared with the respective control.

### Leaching of KP-UM protein during washing

The leaching of KP-UM from colored hair during washing was evaluated for a total of 20 washing cycles, consisting each cycle of an incubation with a shampoo solution followed by a washing with water. At the end of the 20 washing cycles, 1.79 and 9.4% of KP-UM was leached from the colored hair for the 1 and 10% EtOH formulation, respectively. For the 0% EtOH formulation, no leaching was verified even after 20 washing cycles. Even though the amount of KP-UM leached at the end of the washing cycles was below 10% of the initial amount of protein bound to the hair for the 3 formulations, there were some differences between the 3 conditions. An increase on the amount of KP-UM leaching from the colored hair when increasing the amount of ethanol on the formulation was observed at the end of the washing cycles.

### *In vitro* evaluation of KP-UM protein potential cytotoxicity

The development of new hair care cosmetic formulations needs to be supplemented with safety studies, being crucial that the new formulations do not cause any type of cytotoxicity to the users. In 2003, the 7^th^ amendment of the European Union’s Cosmetics Directive (76/768/EEC) was approved, which stipulated the end of animal testing for cosmetic products in European Union. Since 2009, it was also prohibited the animal testing of cosmetic ingredients (Cosmetic Regulation (EC) 1223/2009). Continuous efforts have been done to find alternative approaches capable to replace animal tests by others that give the same information about possible compound’s toxicity^22^.

The potential cytotoxic effect of cosmetic products can be evaluated *in vitro* using appropriate cell lines. Considering the guidelines for testing of new cosmetic products, the NCTC-2544 human keratinocytes cell line was chosen to evaluate the potential cytotoxicity of KP-UM protein. The results of potential cytotoxicity, evaluated in terms of metabolic viability after 24 and 48 h of incubation with the KP-UM protein, are presented in Supplementary Fig. S3.

No signs of evident cytotoxicity were verified at the end of 24 h of incubation, for any of the concentrations of protein tested. At the end of 48 h of incubation there was a trend on cell viability which was dependent on protein concentration, with a decrease on cell viability when increasing the concentration of KP-UM protein. This protein could be considered safe to be incorporated in cosmetic formulations since the cell viability remained always higher than 85%.

### Molecular dynamics of KP-UM binding to keratin filaments

The alignment of the structures of the UM protein with and without the KP peptide and the (GA)_5_ linker, were obtained after 50 ns in water simulations (See Supplementary Fig. S4a). It is visible an almost complete superposition of both β-barrels pointing that the insertion of the KP peptide and the linker sequence do not have significant impact in the UM conformation. These data support the results obtained by CD (Fig. 1b,c). A simulation of KP-UM protein in water and in the formulation with 10% v/v ethanol, was also performed to study the effect of formulation on KP-UM structure. The alignment of both structures (See Supplementary Fig. S4b) show that the formulation does not affect the structure of the protein as no significant differences between the two structures are visible.

**Figure 7:**
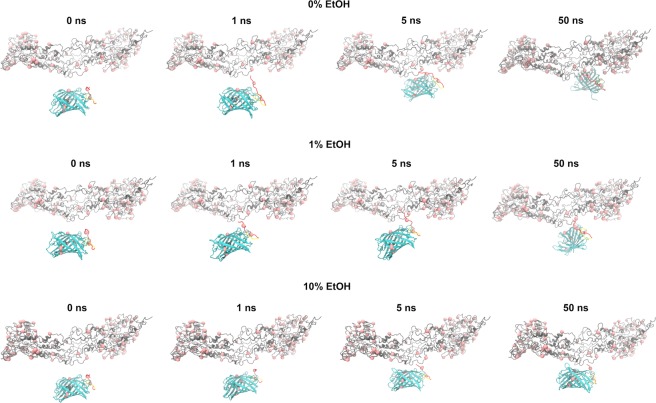
Simulation snapshots of 50 ns of the interaction between KP-UM (cyan) and keratin protofibril (grey). The red spheres represent cysteine residues capable to form disulfide bonds.

In order to study the role of KP and the effect of ethanol from the formulation in the interaction of KP-UM with the keratin filaments, molecular dynamics in different conditions were performed. In Fig. 7, are visible the snapshots for 0, 1, 5 and 50 ns of the simulation with the KP-UM protein and a keratin protofibril. The visual inspection of the simulations clearly shows that the beginning of the interaction between these two proteins is always mediated by the KP sequence added to the UM N’ terminal. Visually, it is also possible to notice that higher concentrations of ethanol difficult the interaction on the KP sequence with the keratin protofibril. The average number of hydrogen bonds between KP-UM and keratin protofibril was determined for the last 10 ns of simulation (See Supplementary Fig. S5). The increase of ethanol decreased the number of hydrogen bonds measured for the interaction: 3.8 ± 2.47, 3.22 ± 1.24 and 1.58 ± 0.742 for 0% EtOH, 1% EtOH and 10% EtOH, respectively.

The determination of the binding energies between the KP-UM protein and the keratin filaments were also determined at the end of 50 ns of simulation and shown to be inconclusive. The interaction between the KP-UM and the keratin is mainly governed by disulfide bonds involving the cysteine residues present in KP sequence of KP-UM and in the keratin^15^. However, this S-S bonds cannot be simulated using the current force fields and the determination of the binding energies does not represent all the interactions. Despite that ethanol seems to decrease the formation hydrogen bonding, ethanol is known to enhance swelling of keratin^15^ and to promote the fixation cysteine containing peptides as previous reported^15^.

## Discussion

A fusion protein, with the capacity to color and improve hair’s mechanical properties, was designed and obtained considering the sequence of the ultramarine chromogenic protein (UM) and a sequence from a keratin-based peptide (KP). A linker constituted by 5 repetitions of glycine and alanine (GA)_5_, was included to increase the structural mobility of UM protein and KP peptide.

To study the effect of temperature on the secondary structure of KP-UM protein, four temperatures (25, 37, 50 and 75 °C) were tested. A structure with a high content of β-sheets, proved by the characteristic maximum and minimum peak at 197 and 220 nm^23^, was verified for all the temperatures. Although, at 75 °C, it was observed an alteration in protein structure proved by the decrease in α-helix and β-strands content and increase in the unordered content. This change in protein structure could be due to partial protein unfolding caused by higher temperatures. Considering a future application in hairdressing salons, the structural similarities observed at 25, 37 and 50 °C (Fig. 1c), allowed us to select the room temperature for the binding assays of KP-UM protein to OB Asian hair.

When KP-UM protein was dissolved in the different formulations, two absorption peaks at 525 and 585 nm were observed on the proteins’ absorbance spectrum (Fig. 2a). These two absorption peaks, corresponding to different protonation states of KP-UM chromophore, were predictable since this protein is derived from a GFP-like protein. The *cis* conformation corresponds to the deprotonated state (anionic – 585 nm) while the *trans* conformation corresponds to the protonated state (neutral – 525 nm)^23,24^. Comparing the spectra of KP-UM protein in the different formulations (Fig. 2a), a shift for lower wavelengths in the absorption peaks was observed as the percentage of ethanol increased. This shift for lower wavelengths with a decrease of solution polarity and solvation promoted by water could be associated with a solvatochroism effect. Solvatochroism is the change of color of a molecule as a function of solvent polarity and dielectric properties. Since more polar solvents (0% EtOH > 1% EtOH > 10% EtOH) gave rise to a red-shift, the KP-UM protein is classified as positively solvatochromic. It was also observed that the peak of the anionic form (585 nm) was more dependent on the solvent since it changed the maximum absorption peak in 10 nm, while the neutral form only changed 5 nm when in the presence of 10% ethanol. This behavior was already described for other GFP-like proteins, where only the anionic peak changed with the solvent ionic strength while the neutral peak remain almost unchanged^25^. Also, differences in the ratio between the two absorption peaks were verified, and this equilibrium seems to be ruled by a hydrogen bond network that allows the proton transfer between the chromophore and neighboring side chains^26^. Analyzing the effect of pH on KP-UM absorbance spectra (Fig. 3b), it was verified that an increase in pH benefit the conversion of *cis* into *trans* conformation, both of them showing similar absorbance values for pH values higher than 8. Moreover, the data suggests that the neutral form (525 nm, *trans* and protonated conformation) of the chromophore is favored at higher pH (pH 8–11), while the anionic form (585 nm, *cis* and deprotonated conformation) dominates at lower pH (pH 3–6). This finding was unexpected since acidic pH should favor the protonated state and more basic pH should favor the deprotonated state. In this way, it was observed a reverse chromophore protonation where acidic pH favored the deprotonated state. This mechanism was already observed for mKeima, a red fluorescent protein derived from the GFP family^27^.

Although the KP-UM protein was based on the sequence of UM protein, a monomeric and non-fluorescent chromogenic protein^7^, the protein showed some fluorescence. In this work, the UM sequence was fused with a keratin-binding peptide (KP)^28^ and a (GA)_5_ linker to promote the binding of the protein to the hair cuticle and to the cortex improving the coloration process. Despite the UM protein is described as chromogenic and non-fluorescent, we found that both, UM and KP-UM proteins, had the ability to convert between non-fluorescent and fluorescent states by irradiation with a specific wavelength of light, a phenomenon often referred to as photoswitching^29,30^. This spectral property was first identified in the *Anemonia culcata* chromoprotein and its mutants^31^. When the KP-UM was excited at ${{\rm{\lambda }}}_{Abs}^{max}$– 525 and 585 nm – no significant emission was observed (see Supplementary Fig. S1). However, when the protein was excited at 440 nm a broad emission spectrum was obtained (Fig. 3c). The photoswitching behavior of UM and KP-UM proteins is currently being studied in more detail and will be subject of a future publication. However, taking in consideration the photoswitching studies performed in GFP or GFP-like proteins, we believe the key event in the switching process is a cis-trans isomerization of the chromophore. This phenomenon is accompanied by changes in the immediate chromophore environment and by distinct protonation equilibria of the chromophore^31^.

Despite the KP-UM protein was able to color the OB Asian hair in all tested conditions (Fig. 4), the hair color and the amount of protein bound to the hair were dependent on the formulation. We were expecting a higher binding efficiency for the formulation with 10% ethanol, related with a higher degree of hair swelling promoted by the ethanol. However, the best result was observed for the formulation with 1% ethanol. This could be related with the swelling of hair shafts and with the protein conformation (exposure of KP peptide to the hair surface) in the formulation with 1% ethanol.

To evaluate the capacity of KP-UM to color hair, CIELAB color measurements (Fig. 5) were performed on hair samples treated with the formulations and on hair samples treated with the formulations and the KP-UM protein. A decrease was observed in the checksum of K/S values for the OB hair incubated with the three formulations without the KP-UM protein. This reduction on the K/S values could be related with the loss of some melanin from the cortex of the damaged hair fibers during the incubation with the formulations. For the OB hair treated with the protein, the highest K/S checksum obtained was for the hair colored with KP-UM in the 10% EtOH formulation. This increase could be related with the effect of ethanol concentration on the color of KP-UM protein or with the swelling of the hair fiber which promotes the binding and penetration of KP-UM into the hair cortex^18^. Comparing the CIELAB differences of all the samples treated with the KP-UM protein with the overbleached Asian hair (Fig. 5b), a darker, reddish and less yellow color was verified, due to the addition of the chromogenic protein. This diagram corroborates the color differences observed for the hair treated with the KPUM protein in the different formulations.

The protein localization and penetration degree into the hair fiber was also assessed by optical (Fig. 6 – Panel A) and by confocal microscopy (Fig. 6 – Panel B). The higher intensity observed in all the samples, compared to the No KP-UM sample, demonstrated the capacity of KP-UM protein to bind to other areas of the hair besides the hair cuticle. Nevertheless, there was a preferential binding of KP-UM in the first 10–15 µm of the hair fiber, corresponding to the cuticle and initial part of the cortex^32^.

All the formulations tested have the capacity to improve the mechanical properties of hair, namely the Young’s Modulus and the Tensile Strength (Table 1). The differences observed between the different conditions (KP-UM in the formulations with 0, 1 and 10% EtOH) could be related with the swelling of the fiber promoted by the ethanol; the amount of KP-UM protein bound; the degree of penetration into the hair cortex; the preferential localization of the protein in the hair fiber and with potential mechanisms of stabilization^15^ of the KP-UM with the keratin proteins of hair.

The leaching of KP-UM protein from the hair was evaluated for a total of 20 washing cycles. It was verified that higher percentages of ethanol in the formulation lead to higher protein leaching from the colored hair. These differences could be associated with the binding of KP-UM protein and with the protein localization in the hair fiber. For example, for the formulation with 10% ethanol, the majority of KP-UM was leached during the first washing cycle, while for the formulation with 1% ethanol, the leaching was only after eleven washing cycles. This might indicate that the protein that was leached during the first washing cycles was predominantly located at the surface of the hair fiber while the remaining protein was trapped inside the inner hair fibers and cannot came out easily to the washing solution^33^.

The molecular dynamics of KP-UM interacting with the keratin filaments (Fig. 7) showed that the KP sequence seems to act as a hook, which when finds the keratin protofibril promotes the junction of both proteins. In addition, this sequence has one cysteine residue that should be accessible to make disulfide bonds with the many cysteine present in the keratinaceous fiber. For all the simulated systems the interaction between the KP-UM protein and the keratin protofibril begun through the KP sequence, and after 5 ns the UM barrel adhered completely to the surface of the keratin protofibril. The effect of the different formulations on KP-UM interaction with the keratin filaments showed that higher concentrations of ethanol can difficult the interaction between the proteins. The hindering effect of ethanol could be related with the number of hydrogen bonds formed between the KP-UM protein and the keratin protofibril, since a reduction in the number of hydrogen bonds was observed when increasing the ethanol concentration.

These results support the differences obtained in the leaching assay during washing, where a higher leaching of KP-UM protein was verified with increasing the percentage of ethanol in the formulation. In this way, the higher bounding stability observed when the hair was colored by KP-UM dissolved in the phosphate buffer could be due to the absence of ethanol in this formulation.

In conclusion, in this study we investigated the capacity of KP-UM protein (Ultramarine protein conjugated with a keratin-based peptide - KP) to simultaneously color overbleached Asian hair and recover hair’s mechanical properties. The KP-UM protein was able to color the overbleached Asian hair, being the hair color obtained mainly dependent on the polarity of the formulation where the protein was solubilized. The binding to the hair cuticle and penetration into the cortex supports its ability to color the hair. Additionally, molecular dynamic simulation studies demonstrate that the interaction of KP-UM with hair is mediated by the KP sequence, proving the importance of this peptide on the ability of KP-UM to bind and color the hair. The KP-UM protein in the three formulations (0, 1 and 10% EtOH) was able to induce a significant recovery of overbleached Asian hair tensile strength and stiffness. Also, the KP-UM was strongly bound to the hair since almost no protein was leached after 20 washing cycles, with a maximum of 9.4% of protein leaching observed for the 10% EtOH formulation. Considering the safety of KP-UM protein when tested in human keratinocytes, we believe that the use of this protein for hair coloration has a great potential.

In this work, we unravel the potential of fusion proteins as complex systems to be incorporated in hair-care products. Chromogenic proteins can be excellent candidates for the design and development of new eco-friendly products for hair coloration. The chromogenic nature of these class of proteins can be also explored in future in textile industry and in the development of new color-based sensors.

## Experimental Section

### Materials

Asian black hair samples were provided by International Hair Importers & Products Inc. (Glendale, New York U.S.A.). KP-UM gene was synthesized by GenScript and cloned in the pET-28a(+) plasmid. The amino acidic sequence of KP-UM protein is presented in Supplementary Table S1. Nickel Magnetic Beads for protein purification were available from GenScript. Molecular weight GRS Protein Marker Blue standards and culture medium were purchased from Grisp, Portugal. All other reagents were acquired from Sigma-Aldrich, Madrid-Spain.

### Expression and purification of KP-UM

*Escherichia coli* BL21(DE3) containing the pET-28a(+)::KP-UM vector was used for KP-UM expression in Terrific Broth – Auto Induction Medium (TB-AIM). A single colony harboring the plasmid was inoculated into LB medium supplemented with kanamycin (kan) and grown overnight at 37 °C. A calculated volume of the pre-inoculum was inoculated into TB-AIM_kan_ and the culture was grown for 24 h at 37 °C, 200 rpm. Cells were harvested by centrifugation at 7000 g, at 4 °C, for 5 min. The cells were resuspended in phosphate buffer (20 mM sodium phosphate, 500 mM NaCl, pH 7.4) with 10 mM of imidazole and protease inhibitor and were lysed by sonication (40% amplitude, 3.0 s on plus 9.0 s off for a total of 10 min on) in a sonicator vibracell^TM^ SONICS. The suspension was centrifuged at 10.000 g, for 40 min, at 4 °C, to separate the soluble fraction. The protein present in the soluble fraction was purified using Nickel magnetic beads with specificity to the His-tag sequence present in the N-terminal of the protein. KP-UM purity was assessed by SDS-PAGE and the purified protein solution was dialyzed against distilled water.

### Characterization of KP-UM protein

#### SDS-PAGE analysis

Protein size and purity were analyzed by SDS-PAGE. The lyophilized proteins were solubilized in ultra-pure water, mix with loading buffer and loaded on 12% SDS-PAGE and stained with Coomassie Blue.

#### MALDI-TOF mass spectrometry

Mass/charge of KP-UM was verified by MALDI-TOF using sinapic acid as the matrix ( ≥ 99.5%). The mass spectra were obtained using an Ultra-flex MALDI-TOF mass spectrophotometer (Bruker Daltonics GmbH) equipped with a 337 nm nitrogen laser. A double layer deposition was used to analyze KP-UM protein. For this, a saturated solution of sinapic acid in ethanol was deposited in the ground steel plate and let dry. The KP-UM protein, previously dissolved in TA30 (30% acetonitrile/70% TFA), was mixed (1:1) with a saturated solution of sinapic acid in TA30. A volume of 2 µL was spotted into the ground steel target plate (Bruker part n° 209519) and analyzed using the reflective positive mode^34^.

#### Circular dichroism (CD) spectroscopy

The effect of temperature in the structure of KP-UM protein was studied by CD spectroscopy using a Jasco J-1500 spectropolarimeter equipped with a temperature controller, and a path-length cell of 1 mm. Four temperatures, 25, 37, 50 and 75 °C, were tested and the same protein concentration was used for all the conditions. The spectra were obtained over the wavelength interval of 185–260 nm at a scan speed of 20 nm/min and bandwidth of 1 nm. Each condition was analyzed in triplicate and the average was used to obtain the final spectra^34^.

#### Bleaching process

The bleaching of black Asian hair was performed according Tinoco *et al.,* 2018^21^ The hair tresses were washed before and after treatment with a classic commercial shampoo (Pantene Basic).

#### Hair treatments with KP-UM protein

Coloring treatments with the KP-UM protein were applied to the overbleached (OB) Asian hair. For the treatments, 0.2 g of OB Asian hair was incubated with 10 mL of KP-UM formulation (2 mg/mL), for 30 min, at room temperature, and dried for 20 min at 60 °C. This process was repeated three times. Three formulations were tested: 0.1 M phosphate buffer, pH 7, (0% EtOH formulation); 0.1 M phosphate buffer pH 7 with 1.5% (v/v) propylene glycol, 0.5% (v/v) benzyl alcohol and 1% (v/v) ethanol (1% EtOH formulation); 0.1 M phosphate buffer pH 7 with 1.5% (v/v) propylene glycol, 0.5% (v/v) benzyl alcohol and 10% (v/v) ethanol (10% EtOH formulation). Hair samples incubated with the formulations without the KP-UM protein, were used as controls. All samples were thoroughly washed in tap water with shampoo and dried at room temperature after treatment to remove loosely bound KP-UM protein.

#### Color strength determination

Color differences in the hair samples colored with the KP-UM protein, were determined using a reflectance-measuring apparatus (Spectraflash 600; Datacolor) according to the CIELAB color difference concept. The influence of the treatment on hair color was assessed in terms of K/S values and chromaticity coordinates. K/S is the Kubelka-Munk relationship, where K is an adsorption coefficient and S is a scattering coefficient. The data were the sum of all K/S values obtained at the wavelength range 360–700 nm. All analysis were performed in triplicate at 3 different places on the hair samples and the data presented are the mean values of these measurements^35^.

#### Bright-Field, fluorescence and confocal microscopy

Individual hair fibers were embedded in epoxy resin and transversal cuts (20 µm) were prepared using a microtome (Microtome Leitz, Oberkochen, Germany). Transversal cuts of OB Asian hair with and without KP-UM protein treatment were analyzed by bright-field and fluorescence microscopy using a LEICA DM 500B fluorescence microscope (Leica, Berlin, Germany). Samples were also analyzed in a Confocal Scanning Laser Microscope (Olympus BX61, Model FluoView 1000). No dye was used in this analysis since KP-UM protein showed to be fluorescent. Images were acquired with the program FV10-Ver4.1.1.5 (Olympus) integrated with Line Series Analysis.

#### Evaluation of hairs’ mechanical properties

The effect of KP-UM protein on the mechanical properties of OB Asian hair was assessed following guidelines outlined in ASTM D1145-95 for fiber tensile testing. Tensile tests were performed using a Hounsfield dynamometer H100KS Model and a set of 30 hair fibers with low variability in terms of hair thickness for each condition was selected. Each hair was individually mounted in the tensile jig by means of a paper template with a fixed gauge length of 20 mm and placed in an excicator prior to analysis. A load range of 25N and a speed of 1.5 mm/min were defined as settings for the tensile strength test^36^. For each hair, applied load against extension were recorded and, using an average mean diameter of 70 µm, data were converted to stress (load/unit area) versus strain (% extension). All measurements were made in the middle part of the hair fibers.

#### Molecular dynamics simulations

The molecular dynamics simulations were performed with the Gromacs 4.0.7 software package and the GROMOS 54A7 united-atom force-field^36,37^. Periodic boundary conditions were applied, with the LINCS^38^ and the SETTLE^39^ algorithms constraining the proteins and water bonds, respectively. The time step used was 2 fs, the water was represented by the single point charge (SPC) model^40^ and the temperature of 300 K was maintained by the V-rescale thermostat^41^. The Parrinello-Rahman barostat^42,43^ (with isotropic coupling) was used to maintain the pressure at 1 atm. The neighbor list was updated every 10 steps, the twin-range method (0.8 and 1.4 nm for short and long range cutoffs) was used for treat the electrostatic interactions. The dielectric constant of 54 was used for reaction field correction^44^.

The molecular model of hair keratin protofibril used was previously developed in our group by Egipto Antunes, and showed to be useful in the study of some compounds effects in the hair fiber^45,46^. The KP-UM model was obtained through structure homology of UM with the KP peptide and the GA linker amino-acid sequence with the X-ray model of the Rtms5 protein (PDB code: 3vk1) using the SWISS-MODEL web service (https://swissmodel.expasy.org).

The software VMD (Visual Molecular Dynamics)^47^ was used to render the simulations snapshots and videos.

#### Simulations protocol

All the simulated systems were composed by proteins (KP-UM or KP-UM and keratin protofibril) and solvent (water or formulations composed of water (97 or 88% v/v), ethanol (1 or 10% v/v), benzyl alcohol (0.5% v/v) and propylene glycol (1.5% (v/v)). The systems were first energy minimized applying the steepest descent method for 2000 steps. Later three short preparatory simulations of 500 ps, were ran to equilibrate the systems. In these runs positions restrains, with a harmonic force of 10^3^ kJ/mol.nm^2^, were applied to all heavy atoms of non-solvent molecules for the first two runs, and only applied to protein alfa-carbons in the third run. The first equilibration simulation was performed in the NVT ensemble (number of particles, volume and temperature constant). The second, third and the production runs were simulated in the NPT ensemble (number of particles, pressure and temperature constant). After equilibration simulations, were performed the final production simulations, generally without positions restrains, by around 50 ns. All systems were simulated in triplicate. Due to the big size of the systems composed by KP-UM and the keratin protofibril, two of the three replicas were ran with positions restrains in the alfa-carbons of the keratin, allowing the simulation of smaller systems boxes. To test if the application of the positions restrains in these systems influence the interaction between the KP-UM and the keratin, one big system without restrains was also simulated.

The hydrogen bonds between KP-UM and the keratin protofibrils were determined using g_hbond tool from GROMACS, with average hydrogen bonds determined for the last 10 ns of interaction between the proteins.

Steered simulations were performed to calculate the binding energy between the KP-UM and the keratin fiber in the different formulations (0, 1 and 10% EtOH). The simulation protocol was the same, with the exception that in the production run (around 5 ns of simulation) was applied a harmonic force of 1000 kJ.mol^−1^.nm^−2^ to the center of mass of KP-UM alpha carbons, and the alpha carbons of keratin fiber were restrained. This leads to the complete separation of the KP-UM from the keratin. The resulting trajectories allowed the generation of different configurations along the Y coordinate (in which the distance between the two proteinaceous structures increases) to perform umbrella sampling simulations^48^ and later calculate the potential of mean force (PMF) of the crossing process using free weighted histogram analysis (g_wham tool of GROMACS)^48–50^. Each umbrella configuration was simulated by 2 ns, with the pulled KP-UM restrained. The position of the KP-UM differs around 0.05 nm from the neighboring configurations. The obtained PMF variation graph allowed the calculation of binding energy, which corresponds to the energy at the final state minus the energy at the initial state.

#### Statistical analysis

Data are presented as average standard deviation (SD), n = 3. Statistical comparisons were performed by one-way ANOVA with GraphPad Prism 5.0 software (La Jolla, CA, USA). Tukey’s post hoc test was used to compare all the results between them, and a Dunnet’s test was used to compare the results with a control. A p-value < 0.05 was considered to be statistically significant.

## Supplementary information

Supplementary Information
