# Supplementary material for: Fusion proteins with chromogenic and keratin binding modules
Source: Sci Rep. 2019 Oct 1;9:14044. doi: 10.1038/s41598-019-50283-0 (PMC6773707; doi:10.1038/s41598-019-50283-0)
Supplement: Supplementary file 1 — Supplementary Information [file 41598_2019_50283_MOESM1_ESM.pdf]

Supplementary Information

Fusion proteins with chromogenic and keratin binding modules

Ana Tinoco[a], Egipto Antunes[a], Madalena Martins[a], Filipa Gonçalves[a], Andreia C. Gomes[b], Carla Silva[a], Artur Cavaco-Paulo[a], Artur Ribeiro[a]**

[a] CEB - Centre of Biological Engineering, University of Minho, Campus de Gualtar, 4710-057 Braga, Portugal

[b] Centre of Molecular and Environmental Biology (CBMA), Department of Biology, University of Minho, Campus of Gualtar, 4710-057 Braga, Portugal

*Corresponding Authors

Experimental Section

Cytotoxicity evaluation

Culture of human skin keratinocytes cell line (NCTC 2544)

Keratinocytes cell line was used as model to evaluate the KP-UM protein cytotoxicity. This cell line was cultured in Dulbecco's modified Eagle's medium (DMEM), supplemented with 7.5 % FBS, 1 % Glutamine (2 mM) and 1 % (v/v) penicillin/streptomycin solution. The cells were maintained at 37 °C in a humidified atmosphere with 5 % CO_2. Culture medium was replaced every 2 days.

MTT assay

The toxicity of KP-UM protein in cell cultures was assessed using the MTT (3-(4,5-dimethylthiazol-2-yl)-2,5-diphenyltetrazolium bromide) assay. Cells were seeded at a density of 22500 cells/well on a 48-well tissue culture polystyrene plate (TPP, Trasadingen, Switzerland) the day before the experiments. The cells were incubated with different concentrations of KP-UM protein (0.5, 1.0, 1.5 and 2.0 mg/mL), which were selected taking in consideration the concentrations applied to the overbleached Asian hair. Cells incubated with DMSO (30 % of the total volume) and cells without the addition of the compounds were used as controls: control of death and control of life, respectively. The cells were incubated with the protein at 37 °C in a humidified atmosphere with 5 % CO_2, for 24 and 48 h. Cell metabolic activity was assessed using MTT viability assay at the end of each time point. MTT solution was added to each well and the mixture incubated for 2 h at 37 ºC. The formed formazan crystals were then solubilized with ethanol/DMSO (1:1) and the color was measured with a 96-well ELISA plate reader at 550 nm, with the reference filter set to 620 nm in a microplate reader (Spectramax 340PC, Molecular Devices, Sunnyvale, CA, U.S.A.). All MTT assays were repeated independently three times (Tinoco et al., 2018).

Leaching of KP-UM during washing

The effect of water and shampoo in the leaching of KP-UM protein from the hair fibers was assessed during a total of 20 shampoo and 20 water washing cycles. For that, the colored hair samples were incubated with 10 mL of a water solution of shampoo (0.1 % (v/v)) for 3 min under agitation. Then, the same samples were incubated with 10 mL of water for 3 min under agitation. The amount of KP-UM protein loss during the washing process was determined at 585 nm against a calibration curve using different KP-UM concentrations.

Table S1

KP-UM protein sequence and predicted molecular weight. Characters in bold correspond to the KP sequence and the $(GA)_5$ linker is underlined.

KP-UM Sequence	Mw (kDa)
**GGVCGPSPPCITT**<u>GAGAGAGAGA</u>MSVIATQMTYKVYMSGTVNGHYFE VEGDGKGRPYEGEQTAKLTVTKGGPLPFAWDILSPQCQYGSIPFTKYPEDI PDYVKQSFPEGFTWERIMNFEDGAVCTVSNDSSIQGNCFTYHVKFRGTNF PPNGPVMQKKTQGWEPNSERLFARGGMLIGNNRMALKLEGGGHYLCEFK TTYKAKKPVKMPGYHYVDRKLDVTNHNKDYTSVEQCEISIARKPVVA	26.73

Note: the protein final size considering the extra sequence (His-tag) from pet28a(+) vector is 29.049 kDa.

Figure S1

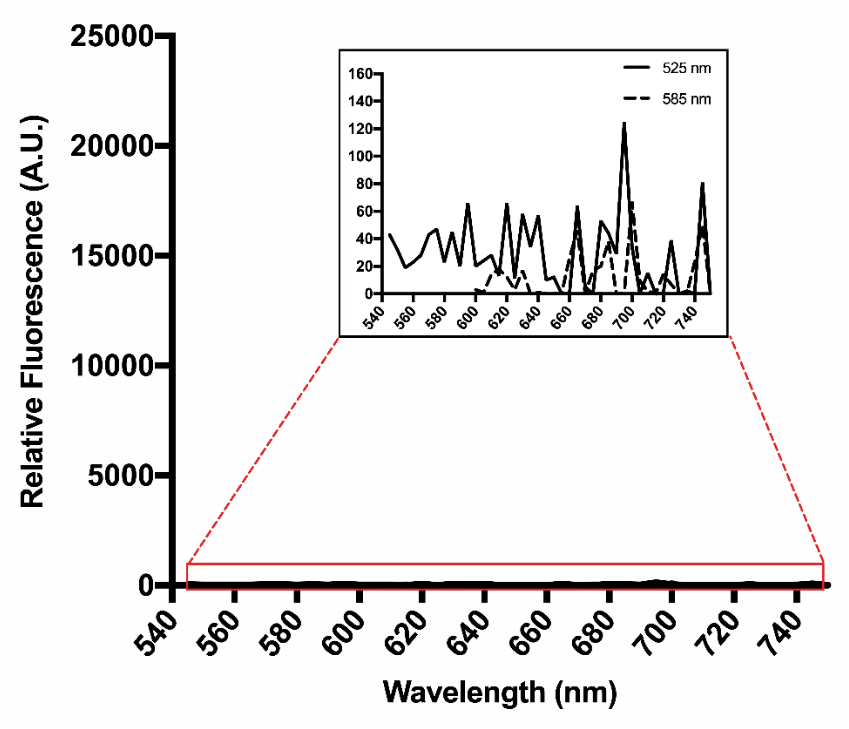

Figure S1. Emission spectra of KP-UM when excited at 525 nm and 585 nm.

Figure S2

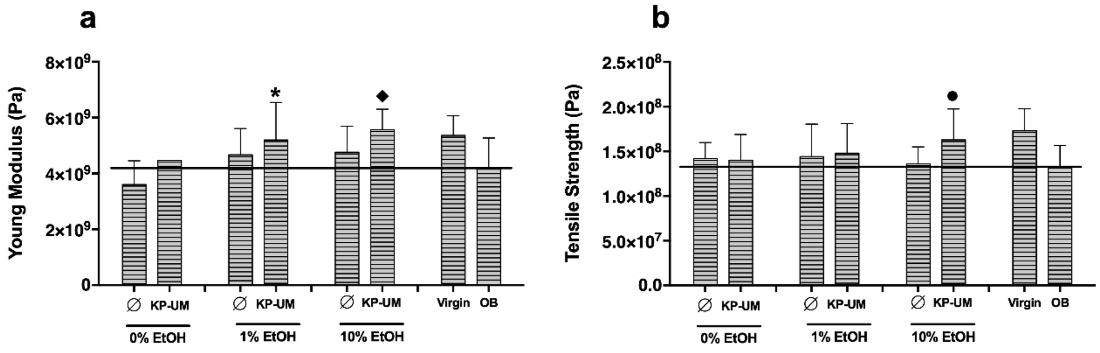

Figure S2. Mechanical resistance parameters: Tensile strength (a) and Young modulus (b) of overbleached Asian hair (OB) before and after treatment with 2 mg/mL of KP-UM protein resuspended in formulations with 0, 1 and 10 % ethanol. Virgin Asian hair was used as control of the effect of bleaching and KP-UM coloration. Values are the mean of SD of twenty independent measurements. Statistical significant differences from the respective control are indicated as: * p-value ≤0.05, ● p-value ≤0.01; ◆ p-value ≤0.001.

Figure S3

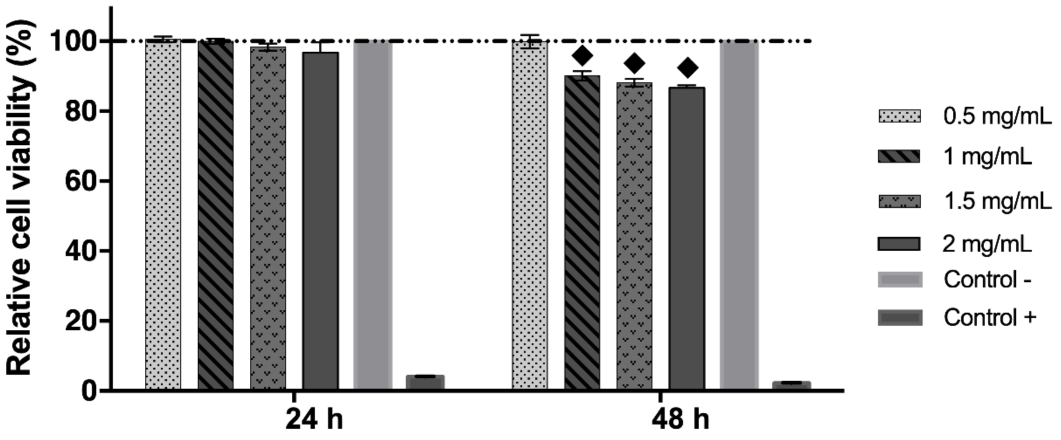

Figure S3. Relative viability of NCTC-2544 keratinocyte cell line evaluated with the MTT assay, after 24 and 48 h incubation with 0.5, 1.0, 1.5 and 2.0 mg/mL of KP-UM protein. Cells incubated with culture medium only were used as life control (Control +) and cells incubated with 30 % DMSO as death control (Control -). Data were determined in relation to the life control. Results are the mean ± SD of triplicate of three independent experiments. Statistical significant differences from the control are indicated as follows: ● p-value ≤0.01; ◆ p-value ≤0.001.

Figure S4

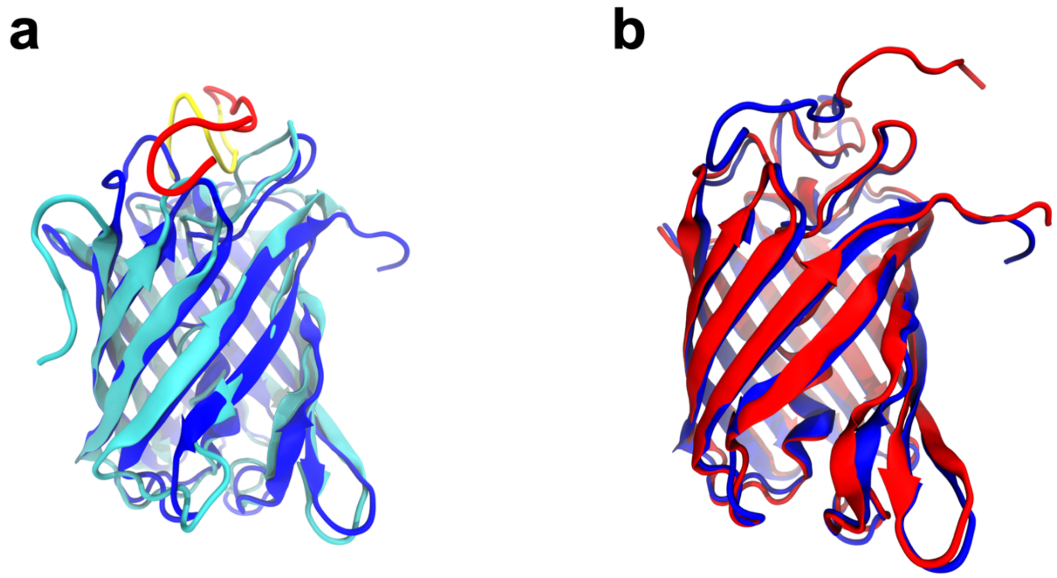

Figure S4. a) Structural comparison between UM (cyan) protein and the KP-UM protein (blue - UM protein; yellow - $(GA)_5$ linker; red - KP sequence) from a lateral (point of view; b) Structural comparison between the KP-UM conformation in water (blue) and formulation with 10 % v/v ethanol (red).

Figure S5

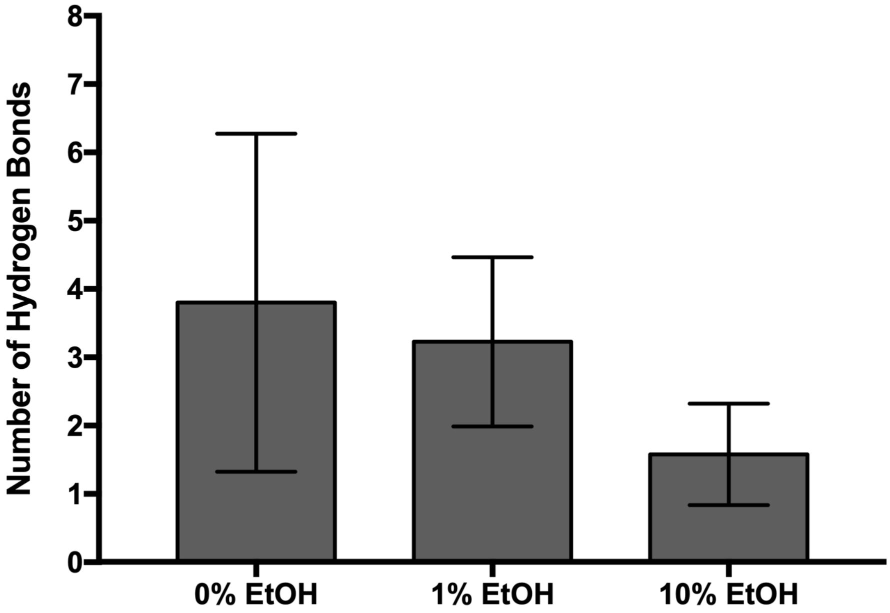

Figure S5. Average number of hydrogen bonds between KP-UM protein and the keratin protofibril, determined in the last 10 ns of simulation.
